# Transient Receptor Potential Ion Channels Control Thermoregulatory Behaviour in Reptiles

**DOI:** 10.1371/journal.pone.0000281

**Published:** 2007-03-14

**Authors:** Frank Seebacher, Shauna A. Murray

**Affiliations:** School of Biological Sciences, University of Sydney, Sydney, Australia; University of Maryland, United States of America

## Abstract

Biological functions are governed by thermodynamics, and animals regulate their body temperature to optimise cellular performance and to avoid harmful extremes. The capacity to sense environmental and internal temperatures is a prerequisite for the evolution of thermoregulation. However, the mechanisms that enable ectothermic vertebrates to sense heat remain unknown. The recently discovered thermal characteristics of transient receptor potential ion channels (TRP) render these proteins suitable to act as temperature sensors. Here we test the hypothesis that TRPs are present in reptiles and function to control thermoregulatory behaviour. We show that the hot-sensing TRPV1 is expressed in a crocodile (*Crocodylus porosus*), an agamid (*Amphibolurus muricatus*) and a scincid (*Pseudemoia entrecasteauxii*) lizard, as well as in the quail and zebrafinch (*Coturnix chinensis* and *Poephila guttata*). The TRPV1 genes from all reptiles form a unique clade that is delineated from the mammalian and the ancestral *Xenopus* sequences by an insertion of two amino acids. TRPV1 and the cool-sensing TRPM8 are expressed in liver, muscle (transversospinalis complex), and heart tissues of the crocodile, and have the potential to act as internal thermometer and as external temperatures sensors. Inhibition of TRPV1 and TRPM8 in *C. porosus* abolishes the typically reptilian shuttling behaviour between cooling and heating environments, and leads to significantly altered body temperature patterns. Our results provide the proximate mechanism of thermal selection in terrestrial ectotherms, which heralds a fundamental change in interpretation, because TRPs provide the mechanism for a tissue-specific input into the animals' thermoregulatory response.

## Introduction

The efficacy of an organism's response to temperature will directly influence its fitness by modulating cellular reaction rates. Hence, animals respond to the spatial and temporal thermal variability in their environment by regulating body temperature [1], [2]. Ectothermic reptiles were the model organisms that established the paradigm of behavioural thermoregulation [3], and most vertebrates engage in some form of thermally inspired behaviour, such as sun or shade seeking. In reptiles, behavioural thermal selection results in body temperatures that fluctuate narrowly around characteristic mean values [4]. The evolution of thermoregulatory behaviour is closely linked to radiation into thermally heterogeneous terrestrial habitats [5]. Hence, selected body temperatures are thought to have coevolved with the thermal sensitivity of performance traits, such as locomotion, growth, or biochemical rates. Accordingly, animals perform best at their mean selected temperatures which may, however, change between seasons, latitudes, populations, and species [6].

Thermoregulation requires the integration between environment, behaviour, and cellular function [2]. It is, therefore, a necessary condition that animals can sense their environment as well as their internal state. The pineal complex may stimulate a thermal response via its secretion of melatonin [7], but it does not act as a tissue specific sensor of temperature. Transient receptor potential ion channels (TRPs) were recently discovered to respond to heat in *Drosophila* and mammals [8], [9], and each of the several known thermo-sensitive TRPs from the vanilloid (TRPV1-4) and melastatin (TRPM8) subfamilies is gated by a characteristic temperature range [10]. TRP ion channel proteins form transmembrane cation permeable channels that depolarise cell membranes by mediating flux of Ca^+^ and Na^+^ [11]. TRPs are associated with free nerve endings of dorsal root ganglion neurons [9]. Unlike other ion channels that show thermodynamically induced small increases in current flow with Q_10_ values of around 2, TRPV1 and TRPM8 respond specifically to temperature with Q_10_ values exceeding 20 [9], [12]. No TRP gene has yet been characterised from a reptile.

Here we test the hypothesis that thermally sensitive TRP genes function in controlling thermoregulatory behaviour in reptiles. Specifically, we aimed to determine, firstly, whether TRP genes are present in reptiles and to resolve their phylogenetic relationship to similar mammalian genes. Secondly, if TRP genes are present in reptiles we aimed to determine in which tissues these are expressed. A temperature sensor can be effective in modifying behaviour only when it is present at the animal surface to sense environmental temperature. In animals with significant internal temperature gradients, it would also be advantageous if sensors were present in the organs to act as an internal “thermometer”. Sensing high temperatures is particularly important for ectothermic reptiles that use basking in the sun as their primary source of heat. Hence, detection of high operative temperatures [13] is essential to choose suitable basking sites as well as to avoid overheating. As out third aim, we therefore tested whether inhibition of TRP proteins would significantly alter thermoregulatory behaviour in a typical reptilian thermoregulator, the crocodile *Crocodylus porosus*.

## Results

### Molecular Phylogeny

**Figure 1:**
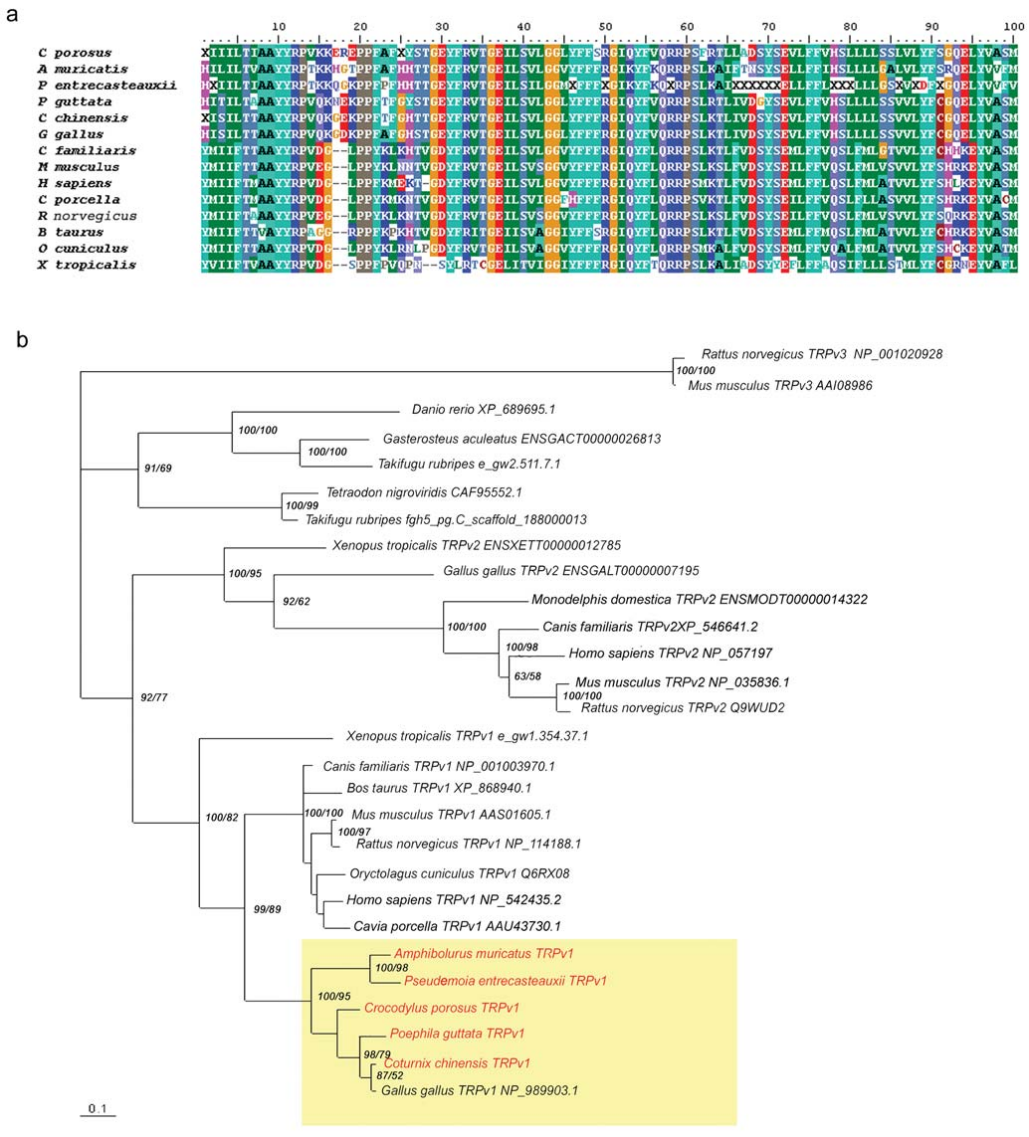
Initial 100 amino acids of the alignment of partial TRPV1 genes, showing a shared indel in the reptilian clade that is absent from the common ancestor, *Xenopus tropicalis.* Fragments were ∼650 bp (*Crocodylus porosus, Poephila guttata, Coturnix chinensis)* and ∼350 bp (*Pseudomoia entrecasteauxii* and *Amphibolurus muricatus*) (a). Phylogenetic relationships of partial TRPV1 and 2 genes based on an alignment of amino acids and analysed using Bayesian Inference (BI). The numbers at nodes indicate posterior probabilities and ML bootstrap values (BI/ML) for clades where these exceed 50%. The new sequences from this work are in red. The reptilian clade is shown in yellow. The full alignment is available from the authors.

We successfully sequenced part of the heat sensing TRPV1 (sensitivity>40^o^C) gene from the crocodile, *Crocodylus porosus*, and two lizards (*Pseudemoia entrecasteauxii* [Scincidae], and *Amphibolurus muricatus* [Agamidae]). To place reptilian TRPV1 gene sequences into a broader phylogenetic context, we also sequenced part of the gene from two birds, the zebrafinch (*Poephila guttata*) and the common quail (*Coturnix chinensis*). We used our novel gene sequences together with existing sequence data to construct a molecular phylogeny of the TRPs (Fig. 1). The TRPV1 sequences of the Reptilia form a well supported (100% and 95% based on Bayesian Inference (BI) and Maximum Likelihood (ML), respectively) sister clade to the mammalian TRPV1 clade, and there is strong support (100/82%) for *Xenopus* as a common ancestor (Fig. 1B). The reptilian grouping is also supported by a distinctive insertion of two amino acids that is not present in either *Xenopus* or mammals (Fig. 1A). Within the Reptilia, archosaurian (crocodiles and birds) TRPV1 form a clade that is distinct from lepidosaurian (lizards) sequences, which branch together with 100%/98% (BI/ML) support (Fig. 1B). The putative fish TRPV1 sequences form a unique clade that is ancestral to both the TRPV1 and TRPV2 genes in tetrapods.

**Figure 2:**
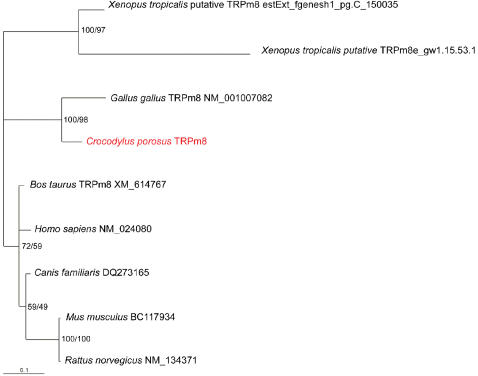
Phylogenetic relationships of TRPM8 genes based on an alignment of amino acids, analysed using BI. The numbers at nodes indicate posterior probabilities and ML bootstrap values (BI/ML) for clades, where it exceeds 50%. The new sequence from this work is in red, and it was ∼500 bp. The full alignment is available from the authors.

We obtained a sequence for the cool sensing [10–25°C; 14] TRPM8 from the crocodile only. Nonetheless, the phylogenetic pattern of the TRPM8 gene is similar to that of TRPV1, with the crocodile and bird sequences forming a strongly supported (100%/98%) sister clade to the mammalian sequences (Fig. 2).

### Gene Expression

**Figure 3:**
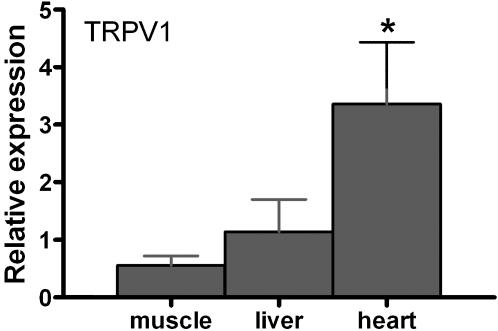
TRPV1 mRNA is expressed in dorsal surface muscle, liver, and heart tissues. Relative expression is significantly greater in heart than in muscle or liver.

TRPV1 mRNA is expressed in heart muscle in all reptiles we considered. In the more detailed analysis of the crocodile we found the gene expressed in surface muscle (transversospinalis complex), liver, as well as heart (left ventricle; Fig. 3). Relative expression is significantly higher in heart than in skeletal muscle or liver (F_2,20_ = 10.44, p<0.001). Additionally, TRPM8 mRNA is expressed in the crocodile heart, muscle, and liver. We did not quantify expression of this gene because levels of expression were extremely low which rendered RT-PCR results unreliable.

### Thermoregulatory Behavior

**Figure 4:**
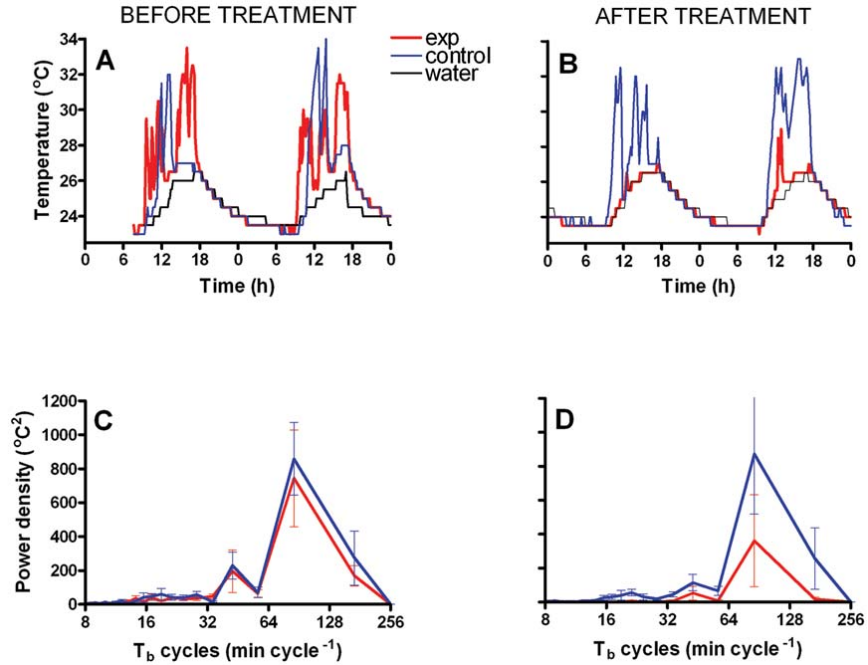
Before treatment, both control (blue lines) and experimental (red lines) animals performed characteristic shuttling behaviour between heating and cooling environments, resulting in periodic oscillations in body temperature (a). After administration of capsazepine, the body temperature of experimental animals ceased to oscillate periodically, while body temperature patterns of control animals remained unchanged (b). Both (a) and (b) show representative examples of data from the same pair of animals. Body temperature time series were transformed into a frequency domain, and the periodogram of the control and experimental animals before treatment shows regular body temperature cycles at 43 and 86 min cycle^−1^ (c). After treatment, these regular cycles disappear when TRPV1 and TRPM8 were blocked, but there is no change in control animals (d). Means from all animals±s.e. are shown in c and d.

*Crocodylus porosus* displays typical patterns of behavioural thermoregulation which consist of periodic movement between basking under a radiant heat source and diving in water [13]; [15]. Paralleling movement patterns, body temperature cycles between water temperature (here 22–26°C) and operative temperatures during basking (here 40–45°C) without reaching either extreme, except at night in the absence of the radiant heat source when animals cool to water temperature (Fig. 4a). After we administered the highly specific TRPV1 and TRPM8 blocker capsazepine, the regular shuttling behaviour between basking and the water ceased, and the animals' body temperature coincided with water temperature except for occasional excursions (Fig. 4b).

**Figure 5:**
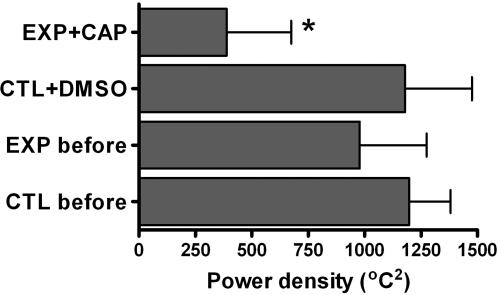
The total power density, i.e. the intensity of periodic body temperature cycles, is significantly less when TRPV1 and TRPM8 were blocked, compared to the other treatments which do not differ significantly from each other (CTL = control animals, EXP = experimental animals, DMSO = DMSO treatment, CAP = capsazepine treatment; means from all animals±s.e. are shown).

Thermoregulatory behaviour is characterised by regular movement between heating and cooling environments. Blocking of the putative thermal sensors could result in random movement between heating and cooling and, hence, random hot or cool body temperatures. Therefore, we analysed the measured body temperature time series for the occurrence of regular, non-random cycles by applying a Fast Fourier transform, rather than comparing absolute body temperature values. Body temperature of control and experimental animals before treatment cycles regularly with peaks at 43 and 86 min cycle^−1^ on average (Fig. 4c). After blocking TRPV1 and TRPM8, power density decreased significantly in the experimental animals, but not in the control animals that received the vehicle (DMSO) only (Fig. 4d). There are no significant differences in power spectra between control animals before injection of DMSO, after injection of DMSO, and experimental animals before injection of capsazepine. However, power densities and, hence, body temperatures cycles of experimental animals decreased significantly after blocking TRPV1 and TRPM8 with capsazepine (main treatment effect: F_1,12_ = 5.09, p = 0.044; before/after control F_1,12_ = 11.71, p = 0.005; interaction F_1,12_ = 8.76, p = 0.012; Fig. 5)

## Discussion

All vertebrates can regulate their internal temperature, or at least possess the capacity to avoid harmful extremes. TRPs are likely to be important in thermoregulation of mammals [12], and our report of homologous genes in reptiles indicates a common ancestry of thermoregulatory systems. Reptiles do have thermally sensitive neurons in their brain [16] although the strongest evidence for a central thermoregulatory control centre in reptiles stems from experiments on the function of the pineal gland and melatonin [17]. Melatonin may act as an intermediary between optical signals, and behavioural and physiological responses [18]. Changes in melatonin levels affect body temperatures selected by some reptiles [19]; [20], but the response is not consistent [7]. Importantly, the pineal/melatonin system does not provide a mechanism of thermal sensation and it cannot explain how the thermal state of different organs is integrated into a cohesive thermoregulatory response.

In mammals and reptiles, the preoptic area of the hypothalamus acts as a thermoregulatory control centre that compares sensory information received from the skin and brain to thermoregulatory set-points [21]–[23], thereby initiating behavioural and physiological responses [24]. However, it is unlikely that the hypothalamus acts as the only integrator of thermal signals, and it is more plausible that several hierarchical control points achieve a precise thermoregulatory outcome [25]. Many reptiles, and particularly crocodiles, regulate body temperature behaviourally [13] and autonomically by cardiovascular adjustments [26]. Additionally, reptiles also respond at a cellular level by modulating muscle performance and anaerobic and oxidative metabolic pathways to compensate for the effects of changing internal temperatures [15]. TRPs can provide the mechanisms by which these multiple responses are integrated. Thermoregulatory responses are elicited by thermal inputs from different regions of the body [25], and in the crocodile at least TRPV1 and TRPM8 are expressed in tissues throughout the body. The potential for local temperature sensation would be particularly important in animals where there are pronounced internal temperature gradients. The hypothesis of a single temperature comparator assumes that internal temperatures are more or less homogenous which is more likely in endotherms with substantial internal heat production than in ‘large’ heliothermic (basking) reptiles. The relative resistances to heat transfer within the body, and between the body and the environment will determine the magnitude of internal gradients of body temperature. Resistance to heat transfer is a function of body mass, as defined by the Biot number, and animal movement in heterogeneous thermal environments [13], [27], [28]. Hence, particularly for ectotherms, it is advantageous to have a thermoregulatory control system that comprises multiple sensors and comparators, particularly because thermal sensitivities may vary between tissues [15].

Thermally sensory neurons that are associated with TRPs are located in the dorsal root ganglia. These neurons project via the dorsal horn interneurons [9], and the thermal response may be controlled centrally via the hypothalamus [29]. In addition to behavioural and cardiovascular thermoregulatory responses [29], sympathetic discharge may also elicit a thermal response directly at the cellular level. Cellular responses to temperature are mediated by β-adrenergic receptors at the cell membrane. For example, metabolic responses may be stimulated by an increase in sympathetic discharge, which will induce increased transcription of the metabolic master controller PGC1α in tissues [30]. Hence, cold can be mimicked by β_3_ and α_1_-adrenergic receptor stimulation [31], which leads to increases in cellular PGC1α mRNA concentrations in rats [30]. Cold induced expression of PGC1α stimulates transcription of metabolic enzymes such as cytochrome c oxidase and F_0_F_1_-ATPase, which are rate limiting steps in oxidative phosphorylation [30]. Hence, metabolic upregulation and acclimation in response to cold could be part of the thermal response elicited by TRPs located in target tissues, although this hypothesis must be tested experimentally. Nonetheless, expression of TRP in heart, liver, and muscle tissue suggests that there is at least a local sensory input. The significant change in body temperature patterns following blockade of TRPV1 and TRPM8 unquestionably links these proteins to control of behavioural thermoregulation in crocodiles. The expression profiles of TRPV1 and TRPM8 indicate functions as a exteroreceptor as well as an enteroreceptor, and a hierarchical model of control [25] seems to be the most appropriate hypothesis considering that different tissues have different thermal tolerances.

Our report that TRPV1 and TRPM8 genes are present in reptiles and show a distinctive sequence pattern, and that they function to significantly affect thermoregulatory behaviour opens a new direction for future research. Significant advances will be made in the field of ectotherm thermoregulation by determining how local sensors, including several others in the TRP family [10], may stimulate cellular responses to temperature change.

## Materials and Methods

### Molecular Phylogeny

Semi-degenerate primers for conserved regions of TRPV1 and TRPM8 were designed from an alignment of mammalian and chicken sequences of these genes present in GenBank. The primer sequences were:

TRPV1a

F 5′ CAAGTGGGACMGAKTTGTCA 3′

R 5′ GTCACYACVGCTGTGGAAAA 3′

TRPV1b

F 5′ CARGACAARTGGGACMGATT 3′

R 5′ TAWATGCCCATCWGCTGRA 3′

TRPV1c

F 5′ TGCCTACTAYMGRCCTGTGC 3′

R 5′ ARCATRTTGAGCAGRAGGATG 3′

TRPM8

F 5′ TGGGGCATGRTYTCCAAC 3′

R 5′ GTTCCAYTCCAGCAGMAGC 3′

PCR reactions contained 200 mM primers and either 1×Immomix (Bioline, USA) or 1 unit Phusion polymerase with the manufacturer's buffer (Finnzymes, Espoo, Finland). Touchdown PCR protocols were used: 95°C for 7 min, 20 cycles of 95°C for 30 sec–1 min, 65°C–63°C for 30 sec–1 min, reducing by 0.3–1°C every cycle to 55°C, 10 cycles at 55°C and 72°C for 2 min. Re-amplification of the PCR products was conducted either by repeating the initial program or by using a program of 95°C for 7 min, 35 cycles of 95°C for 1 min, 58°C for 1 min, and 72°C for 2 min. PCR products were visualized on 2% low melting temperature agarose gels. Bands were exised, purified (Wizard SV Gel and PCR Clean-up System, Promega, U.S.A.) and sequenced (SUPAMAC, University of Sydney, Australia).

PCR products from a crocodile (*Crocodylus porosus*), two lizards (Agamidae: *Amphibolurus muricatus*; Scincidae: *Pseudomoia entrecasteauxii*), and two birds (Passeridae: *Poephila guttata,* Phasianidae: *Coturnix chinesis*) were sequenced (SUPAMAC, University of Sydney, Australia), and submitted to GenBank (accession numbers EF052866, EF052867, EF052868, EF052869, EF061141).

For the phylogenetic analyses, additional sequences were obtained through searches of translated protein sequences on the databases GenBank, Ensembl, and the Joint Genome Institute (JGI) (accession numbers are given in Figs. 1 and 2), and aligned using Clustal W [32]. We used the Akaike information criterion (AIC) to determine the log likelihood of 56 and 79 different substitution models for the analysis of our aligned cDNA and protein sequences, respectively, using the programs Modeltest [33] and ProTest [34]. The optimal models were JTT [35] and gamma distributed across-site rate variation for both the TRPV1/2 protein alignment and the TRPM8 protein alignment. Alignments were analysed using maximum likelihood (ML) and the optimal models and parameters in the program Phyml v2.4.4 [35]. ML bootstrap analyses were performed using 100 replicates. Alignments were also analysed using Bayesian inference (BI) using the same parameters, in the program Mr Bayes [36]. Five hundred thousand generations were run, or until the standard deviation of split frequencies was less than 0.01, and sampled every 10 generations, discarding the results of the first 250000 generations [37].

### Gene Expression

New primers and probes for qRT-PCR were designed from the new partial sequences of *C. porosus* TRPV1, TRPM8 (GenBank Accession no. EF052870) and, as a housekeeping gene, 28S rDNA (GenBank accession no. EF063685). Following behavioural experiments (below), crocodiles were euthanized (sodium pentabarbitone 200 mg kg^−1^) and liver, heart (ventricle), and peripheral dorsal muscle (longissimus complex) samples were collected and immediately stored in RNAlater (Ambion, U.S.A.). RNA extraction, reverse transcription, and qRT-PCR (using an Applied Biosystems 7500 qRT-PCR machine, Applied Biosystems, U.S.A.) were conducted according to published protocols [38], with the following modifications: tissue was homogenized using a rotor-stator tissue homogenizer (Biospec Products, USA), isopropanol precipitation occurred overnight at −20°C, RNA quality was verified on a Bioanalyser (Agilent Biotechnologies, USA), and reverse transcription was performed using Bioscript (Bioline, USA) following the manufacturer's protocol.

Real-time PCR reactions for *C. porosus* genes contained: 1×Immomix, 4–5 mM MgCl_2_, 100–900 nM primer and probe, 1×ROX reference dye (Invitrogen, USA), and ∼50 ng cDNA. The cycle consisted of: 95°C for 7 min, 40 cycles of 95°C for 20 sec, 60°C for 1 min. The primer and probe sequences were:

TRPV1

F 5′ CTCCTGCTGAGCTCTCTGGT 3′

R 5′ ACCGAGTAAATGCCCATCTG 3′

P-TRPv1 (DCY5)CAGGAACTGTATGTGGCTTCCATGG(DBH2)

TRPM8

F 5′ GACCTGGCCAATGATGAGAT 3′

R 5′ CCATTTTCCAGGAAGAGACG 3′

28S rRNA

F 5′ ACGCTTGGTGAATTCTGCTT 3′

R 5′ GGCAGGAGGTGTCAGAAAAG 3′

P-28S (DHEX)CGACGTCGCTATGAACGCTTGG(DBH1)

Gene expression data were collected from 6 individuals and assays were performed in triplicate. Relative TRPV1 gene expression in different tissues was calculated according to Pfaffl [39] using the grand mean of all tissues as the control, and the expression of 28S rRNA as the endogenous reference. Relative expression between tissues was compared by a one-way ANOVA on log transformed data followed by a post hoc Tukey test. Although we detected TRPM8 sequences from re-amplified PCR products, the Ct values for TRPM8 in the RT-PCR assays were consistently too high (>35) to reliably quantify expression levels.

### Behavioural experiments

All procedures were approved by the University of Sydney Animal Ethics Committee (approval #L04/11-2005/1/4234). Juvenile estuarine crocodiles (*Crocodylus porosus*, Schneider; n = 14), were obtained from a crocodile farm (Wildlife International, NT, Australia). Average body mass of crocodiles before experimentation was 172.9±6.7 g and average snout-tail length was 422.0±3.0 mm (mean±S.E.). In the experimental tanks, shelter and water (up to 200 mm depth) were provided at one end, and a dry basking space at the other end. A full spectrum UV light source (ReptiGlo, Australia) was suspended above the tank (10 h L∶14 h D), and an infrared heat lamp delivered 400 W m^−2^ to the dry basking area between 9:00 h–17:00 h. Crocodiles were fed live crayfish (farmed *Cherax destructor*) and insects 3 times per week which closely resembles their natural diet.

During the experiment, pairs of crocodiles (one experimental animal and an independent control [n = 7 pairs total]) were housed in plastic tanks (830×620×1250 mm) which were designed so that animals could thermoregulate behaviourally. We surgically implanted temperature data loggers (iButton, Dallas Semiconductor, U.S.A) into the peritoneal cavity of the crocodiles [13], and animals were allowed to recover for at least 1 week before experimentation. After 4 days of body temperature data recording, the experimental animals were injected with the specific TRPV1 and TRPM8 blocker capsazepine [40; 12 mg kg^−1^, AG Scientific, U.S.A.] dissolved in DMSO (0.2 ml total volume), and control animals received the equivalent volume of DMSO only. The experimental design therefore provided before and after controls, as well as independent controls.

We analysed body temperature data collected for 2 d before-and for 2 d after drug administration, but excluding data from the day when animals were injected to avoid the potentially confounding effect of handling stress. The body temperature time series were expanded to 1024 data points, and Fast-Fourier transforms were conducted in Systat 10.0 software. We integrated the resulting periodograms and used log transformed integrals in the data analysis to meet assumptions of normality; the magnitudes of the integrated periodograms express the intensity of periodic cycles in body temperature. Integrals were compared by ANOVA with Treatment (control/experiment) as factor, and before/after injection of DMSO or capsazepine as a repeated measure. Means were compared by post-hoc Tukey tests, and significance was set at p<0.05.
